# Selective de‐implementation of routine in vivo dosimetry

**DOI:** 10.1002/acm2.13953

**Published:** 2023-03-06

**Authors:** Serena P. H. Mao, Sarah Han‐Oh, Joseph Moore, Ellen Huang, Todd R. McNutt, Annette N. Souranis, Valerie Briner, Aditya Halthore, Sarah R. Alcorn, Jeffrey J. Meyer, Akila N. Viswanathan, Jean L. Wright

**Affiliations:** ^1^ Department of Radiation Oncology and Molecular Radiation Sciences The Johns Hopkins University School of Medicine, Sidney Kimmel Comprehensive Cancer Center Baltimore MD USA

**Keywords:** de‐implementation, diode, in vivo dosimetry

## Abstract

As cone‐beam computed tomography (CBCT) has become the localization method for a majority of cases, the indications for diode‐based confirmation of accurate patient set‐up and treatment are now limited and must be balanced between proper resource allocation and optimizing efficiency without compromising safety. We undertook a de‐implementation quality improvement project to discontinue routine diode use in non‐intensity modulated radiotherapy (IMRT) cases in favor of tailored selection of scenarios where diodes may be useful. After analysis of safety reports from the last 5 years, literature review, and stakeholder discussions, our safety and quality (SAQ) committee introduced a recommendation to limit diode use to specific scenarios in which in vivo verification may add value to standard quality assurance (QA) processes. To assess changes in patterns of use, we reviewed diode use by clinical indication 4 months prior and after the implementation of the revised policy, which includes use of diodes for: 3D conformal photon fields set up without CBCT; total body irradiation (TBI); electron beams; cardiac devices within 10 cm of the treatment field; and unique scenarios on a case‐by‐case basis. We identified 4459 prescriptions and 1038 unique instances of diode use across five clinical sites from 5/2021 to 1/2022. After implementation of the revised policy, we observed an overall decrease in diode use from 32% to 13.2%, with a precipitous drop in 3D cases utilizing CBCT (from 23.2% to 4%), while maintaining diode utilization in the 5 selected scenarios including 100% of TBI and electron cases. By identifying specific indications for diode use and creating a user‐friendly platform for case selection, we have successfully de‐implemented routine diode use in favor of a selective process that identifies cases where the diode is important for patient safety. In doing so, we have streamlined patient care and decreased cost without compromising patient safety.

## 1 INTRODUCTION

Modern external beam radiation therapy (EBRT) utilizes the delivery of high‐energy photon beams directed at a radiographically delineated target within the patient's body. Safe and accurate treatment delivery is dependent on many important factors including treatment planning and dose calculation, reliable patient positioning, and treatment imaging to ensure proper execution of the intended treatment.^1^ Quality assurance (QA) programs have been widely implemented to ensure that the prescription dose accurately matches the dose that a patient receives. Historically, in vivo dosimetry was routinely utilized for QA in patients undergoing conventional radiation therapy to identify major errors in patient setup, dose calculation, or beam parameters (e.g., beam energy or use of wedge).^2^

The diode is one of many types of detectors that have been shown to provide accurate in vivo dosimetry, and the small device can be placed directly on a patient's skin in the beam pathway to measure the dose delivered in that location in real‐time.^3^, ^4^ However, its accuracy may be limited, as the dose captured is a point dose and is sensitive to minor variations in diode orientation and position.^5^ The shadow effect of diode detectors leading to dose reduction in the radiation treatment field has also been previously reported.^6^ As such, it is a tool that can be useful for detection of major errors in patient setup, dose calculation, or treatment beam parameters including beam energy and the use of wedges, but it generally has a wide tolerance range from 5% to 10% without incorporating beam‐dependent and intrinsic correction factors suggested in AAPM Task Group 62, such as wedge, SSD, field size, temperature, angular dependence, etc.^5^ The tighter tolerance is possibly applied with better accuracy achieved with optimal placement of a diode and use of the correction factors, but still up to 5%. While in vivo dosimetry was recommended by the American Association of Physicists in Medicine Task Group for QA in selected patients undergoing radiation therapy in its 1994 report, such recommendations may no longer fully reflect the requirements of modern EBRT.^7^ As cone‐beam computed tomography (CBCT) has become the localization method in many cases, a patient's alignment can be comprehensively verified with 3D volumetric images instead of inferring the setup accuracy from a point dose diode measurement on the skin. Independent verification of the dosimetric accuracy of each treatment plan, including a secondary dose/monitor unit calculation and multiple initial treatment plan/chart reviews, is another standard for good clinical practice which is either strongly recommended or required by multiple accreditation programs, including the Accreditation Program for Excellence (APEx) and the American College of Radiology.^8^, ^9^ This improvement in clinical safety and quality (SQA) standards also contributes to less demand for in vivo diode measurements. Finally, automation in linear accelerator technology, such as record and verify systems and dynamic or motorized wedge placement, allow fewer transcription errors, thus further limiting meaningful diode utilization to selected clinical situations where the aforementioned advancements are not applied.

In our department, diode measurements have been routinely made for all EBRT cases that did not use intensity‐modulated radiation (IMRT) for many years. For non‐IMRT EBRT case, the diode measured dose has served as an integral role to confirm the accuracy of patient setup, plan transferring, and treatment delivery at the first fraction by comparing with the predicted point dose from the corresponding treatment plan. This pattern of routine use had not been re‐evaluated as newer technologies and treatment approaches became more widely employed. In 2020, our SQA Committee recommended an evaluation of diode use, from the perspective that routine use represented a low‐value utilization of the technology and that a more tailored approach would facilitate greater efficiency of patient care without compromising patient safety. We undertook a quality improvement initiative to de‐implement the routine use of diodes in favor of a tailored case selection. We comprehensively evaluated the potential utility of diode utilization in our department, generated a revised policy for diode use based on the initial evaluation, and implemented the policy. Here we report on our experience with this de‐implementation quality improvement project and its impact on diode utilization in the 4‐month period before and after adoption of the new policy.

## 2 METHODS AND MATERIALS

In our department, indications for diode use previously included all non‐IMRT EBRT cases. The initial policy provides the following guidance:

1. Diode measurements are to be made on day 1 for all non‐IMRT cases, and in cases of >5 fractions can be done within the first three fractions if time does not permit use on day 1.
2. If a diode measurement does not agree with the RadCalc sheet, the therapists will page physics for resolution. The tolerances for diode measurements with only SSD correction factor applied are:
3. Within 5% for electrons and photon fields with fewer than three segments.
4. Within 7% for wedge fields.
5. Within 10% for photon fields with three or more segment field.

For electron and photon treatments with 1–5 fractions in total, the diode needs to be removed from the patient's skin before more than 10% of the total dose has been delivered. If the diode reading is out of tolerance, patient setup should be re‐checked and contact the physicist to evaluate the situation before the remainder of the treatment can be delivered.

For cases with >5 fractions, physics is to be contacted for evaluation when a fraction #1 diode reading is observed to be out of tolerance. If no clear cause for the out‐of‐tolerance reading is identified, the therapist must repeat the failing diode on fraction #2 upon advice of physics. If the diode measurement fails again, physics must be contacted and must be present for diode placement on fraction #3.

We convened a working group within our SQA committee to re‐assess the utility of diodes in the current era. The working group carried out the following assessments:

1. A review of the departmental incident learning system to classify cases where diode in vivo measurements had identified treatment errors and would therefore be clinically useful to prevent future errors.
2. A review of current QA practices with consideration of what clinical scenarios these QA practices would make the need for diode measurements superfluous.
3. A review of current workflows and communication patterns regarding diode utilization and an assessment of optimal means of communicating in the setting of revised recommendations for diode use.

After the working group completed its assessment and made recommendations, a new policy for diode use was generated with identification of specific cases in which diodes should be used; this policy was approved and formally implemented on 9/15/2021. We also created a revised dropdown menu for treatment planning approaches in our standardized treatment planning note, which is generated via a web‐based platform. This note communicates to physics and radiation therapy teams that a diode should be used, as well as serving as an order and billing justification. For treatment planning scenarios in which a diode is recommended, an order for diode use and auto‐populated justification language is automatically generated within the treatment planning note.

Following institutional review board approval, we retrospectively reviewed treatment prescriptions for external beam radiotherapy in our radiation oncology database and identified 4459 unique prescriptions and 1038 unique instances of diode in vivo dosimetry between 15 May 2021 and 15 January 2022, representing the 4‐month periods before and after the rollout of the revised diode policy. Variables extracted from the database included: treatment site name, treatment technique (IMRT, 3D conformal, electron, total body irradiation [TBI]), fractionation regimens, and utilization of diodes.

## 3 RESULTS

### 3.1 Review of incident learning system database

A review of all event reports over the last 5 years identified three known cases in which diodes detected measurements that were outside the pre‐determined tolerance out of a total of 13 225 diodes used during this period, and these all occurred in specific treatment scenarios where CBCT was not able to be used: two incorrect electron energy and one source‐to‐skin distance (SSD) error in an extended SSD case.

### 3.2 Review of QA systems that would identify same error type as a diode

A diode measurement can serve as a verification of overall treatment accuracy from the treatment planning system (TPS) through patient setup and treatment. It should be noted that the current diode tolerance, 5%–10%, will catch MU transfer errors that are greater than the tolerance limit. Regarding patient setup accuracy, cone beam computed tomography (CBCT) and portal images can verify a patient set‐up in 3D and 2D geometry, respectively. The visual source‐to‐skin (SSD) check performed by therapists on the first day of treatment is an additional check. Given that a diode is only a one‐point verification of patient set‐up, its utility in catching setup errors for patients using modern 3D and 2D imaging is limited.

Given the low number of deviations found by the diodes and the availability of other QA tools in use, we proposed to limit the use of diodes to specific cases where alternative QA tools are not used or the diode is indispensable for patient safety.

### 3.3 Recommendations for diode use

Based on the above review of safety incidents and currently utilized QA practices, our working group identified the following scenarios in which diodes could identify errors that would not be likely to be detected by other QA processes:

1. When no IMRT QA is performed and CBCT is not used, a diode measurement plus port films of 3D conformal photon fields can provide an additional assurance of accurate patient set‐up.
2. Patient setup for TBI is visually performed with SSD measurements. The diode can provide an overall accuracy of the patient and compensator setup and prescribed dose delivery.
3. Electron treatment either planned in the TPS or clinical setup will benefit from a diode measurement in terms of accurate patient set‐up and dose delivery since the patient set‐up is usually performed visually without portal films or CBCT.
4. Cardiac devices within 10 cm of the treatment field may benefit from diode use. This is a scenario where actual dose delivered at a particular point on the patient is of particular value and may impact risk assessment and management of the device (frequency of interrogations, etc). The group felt this clinical scenario warranted utilization of a diode placed directly over the skin overlying the cardiac device, which is typically very superficial and just under the skin.
5. Specific clinical scenarios may benefit from diode use on a case‐by‐case basis. For example, CBCT imaging may be initially prescribed but found to be not feasible due to inadequate machine clearance around the patient. In such a scenario, diodes could then be utilized as part of setup verification. Additionally, a provider may wish to confirm dose at the patient surface when superficial tissues are at particular risk for residual disease, as may be the case for inflammatory breast cancer treatment or direct skin involvement.

The guidance for response to out‐of‐tolerance readings was unchanged from the initial policy described in materials and methods.

### 3.4 Diode utilization

**FIGURE 1:**
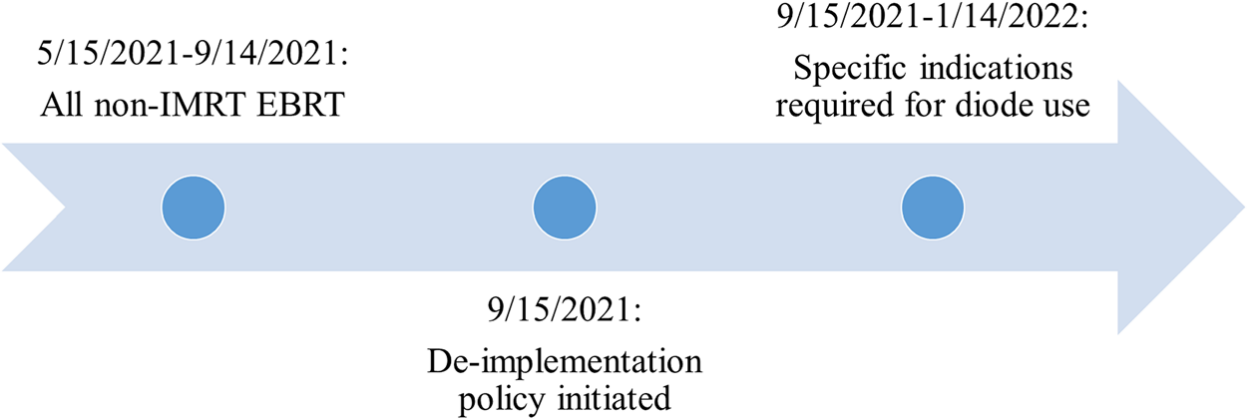
Schema of de‐implementation policy study timeline for diode use. Prescriptions were identified from the 4‐month period prior to initiation of the de‐implementation policy (5/15/2021 to 9/14/2021) and 4‐month period after (9/15/2021 to 1/14/2021). Prior to de‐implementation, diodes were used for all non‐IMRT EBRT. After de‐implementation, diodes were used for specific indications only (see Table 1). EBRT, external beam radiation therapy; IMRT, intensity‐modulated radiation therapy.

The selective de‐implementation policy shifted the use of diodes from all non‐IMRT EBRT cases to specific clinical indications where alternative methods of QA were not available (Table 1). The study period included a 4‐month span (5/15/2021 to 9/14/2021: pre‐policy) prior to and after (9/15/2021 to 1/14/2022: post policy) the initiation of the policy change on 9/15/2021 (Figure 1).

**TABLE 1:** Indications for diode in vivo dosimetry.

Original diode policy	Revised diode policy
All non‐IMRT EBRT cases	3D conformal photon fields when no IMRT (QA) is performed Without CBCT imaging guided setup CBCT imaging isocenters that do not coincide with the treatment isocenter (including extended SSD) TBI Electron beams Cardiac devices within 10 cm of the treatment field Special request for specific clinical scenario

Abbreviations: CBCT, cone‐beam computed tomography; EBRT, external beam radiation therapy; IMRT, intensity‐modulated radiation therapy; QA, quality assurance; SSD, source‐to‐surface distance; TBI, total body irradiation.

**FIGURE 2:**
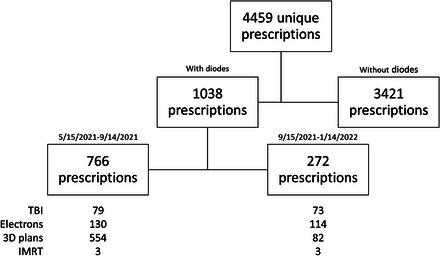
Characteristics of prescriptions during study period (5/15/2021 to 1/14/2022). There were 4459 unique prescriptions during the study period. Of these, 1038 utilized in vivo diode dosimetry for quality assurance (QA) while 3421 did not. Prior to and after the initiation of the de‐implementation policy for diodes on 9/15/2021, there were 766 prescriptions and 272 prescriptions that utilized diodes, respectively. Diode use was mainly observed in TBI, electrons, and 3D plans. IMRT, intensity‐modulated radiation therapy; TBI, total body irradiation.

**FIGURE 3:**
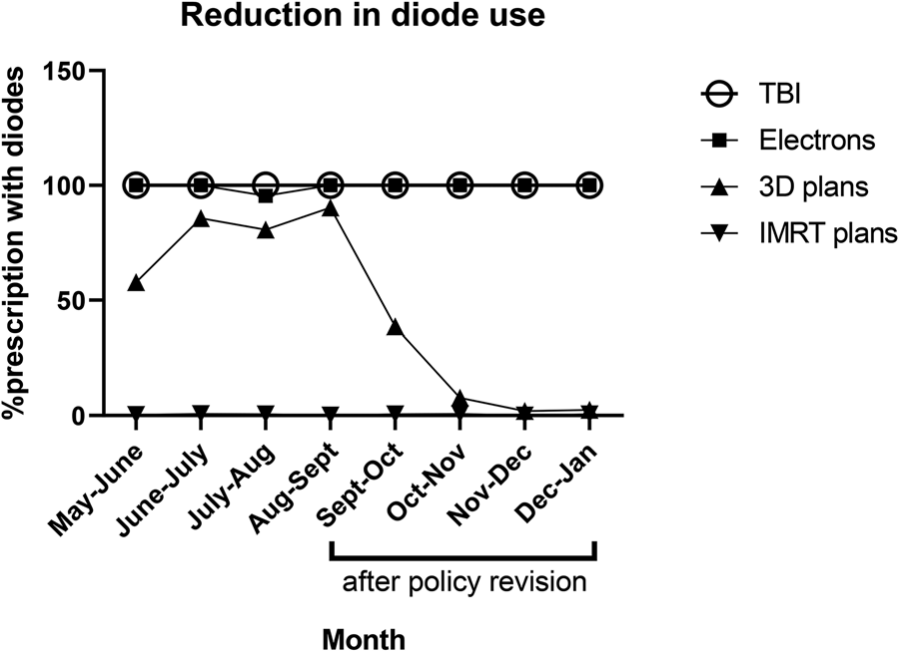
Selective reduction in diode use after de‐implementation policy was initiated. Following the initiation of the de‐implementation policy, all TBI (large circles) and electron (squares) prescriptions continued to use diodes for quality assurance (QA) while use of diodes in 3D prescriptions (up‐right triangles), which routinely utilizes cone‐beam CT imaging, was reduced. Diode use for IMRT prescriptions (downward triangles) remained appropriately low throughout the study period. IMRT, intensity‐modulated radiation therapy; TBI, total body irradiation.

We identified a total of 4459 individual treatment courses, including TBI, electron, 3D conformal, and IMRT courses, across five clinical sites during the overall study period (Figure 2). We observed an overall decrease in the use of diodes from 32% to 13.2% between the pre‐ and post‐policy periods. This reduction was due to a substantial decrease in diode utilization for 3D conformal cases (Figure 3). Prior to the implementing the new policy, 76.5% of 3D conformal treatment courses utilized diodes, and after policy implementation, this proportion decreased to 13.7%. In the last 2 months of the post‐policy period, diode utilization for 3D conformal cases was 3.4%. Diode utilization for TBI, electron, and IMRT cases was consistent between the pre‐ and post‐policy periods, at 100% and 100% for TBI, 0.4% and 0.4% for IMRT, and 99.2% and 100% for electrons, for the pre‐ and post‐policy periods, respectively.

## 4 DISCUSSION

We report on the successful initiation of a policy to de‐implement the routine use of diodes through the employment of a stakeholder‐informed, tailored approach to diode utilization. Review of our records prior to and after de‐implementation demonstrates reduction of diode use for 3D conformal radiotherapy plans, while maintaining appropriate diode procedures for clinical scenarios felt to benefit from diode use for QA, such as TBI and treatment with electrons. To our knowledge, this is the first published paper delineating a de‐implementation strategy for QA in radiation oncology.

Electronic portal imaging devices (EPID) can be utilized to measure exit dose of treatment beams through a patient to detect major treatment errors including treatment machine or patient setup errors.^10^, ^11^ The EPID‐based exit dosimetry has been used as an attractive alternative to traditional in vivo dosimeters including diode, thermoluminescent dosimeters (TLD), or metal oxide semiconductor field effect transistors (MOSFETs) due to several advantages; no additional set up time, no need to be placed on the patient's skin, and versatile use for most of treatment techniques including 3D conformal treatment, IMRT or VMAT. Nonetheless, this advanced in vivo dosimeter is not applicable for certain cases such as TBI or electron treatment. Therefore, our de‐implementation process reported here would be helpful for a safe transition to EPID‐based method as well.

As the field of radiation oncology evolves, more sophisticated approaches to QA continue to be developed. Furthermore, errors in the era of modern radiation therapy planning are often a result of failures in the clinical workflow due to the increasing complexity of treatment planning and delivery. TG100 sought to evaluate the sources of such errors and propose guidelines for risk‐based quality management methods.^12^ While TG100 does not specifically address in vivo dosimetry assessment, the report states that it is crucial to develop a QA program with automated checks of dosimetry, MLC motion, patient setup, and motion that ensures accurate treatment delivery. After the initiation of our de‐implementation policy, diode in vivo dosimetry continues to serve as an integral role to confirm the accuracy of patient setup, plan transferring, and treatment delivery for specific scenarios as previously described. In the patient populations in which diodes were utilized at our institution, the failure modes leading to dosimetric error were identified. Focus was given to the population of 3D conformal patients that utilized CBCT. The failure modes discussed in these cases were either inaccurate setup or incorrect dose distribution. In the case of inaccurate setup, the use of CBCT is more robust in identifying setup errors than diode use. For the case of incorrect dose distribution, we utilize port films for all 3D cases which confirms the shape of the dose distribution, while routine daily, monthly, and annual QA confirms the output of the machine. As the use of diodes in fact causes a dose discrepancy, the use of non‐diode safety mechanisms remedies the failure mode of dose shadowing. Our QA program also incorporates independent verification of the dosimetric accuracy of each treatment plan, including a secondary dose/monitor unit calculation and multiple initial treatment plan/chart reviews, as well as record and verify systems and dynamic or motorized wedge placement through automation in the linear accelerator technology. Furthermore, multiple therapists are carefully monitoring treatments while in progress to minimize delivery errors.

At the same time, we are often reluctant to abandon historical practices that may be superseded by newer approaches. It is widely acknowledged that waste in the US healthcare system contributes to high costs, and that in the United States we are slow to give up practices that provide little or no benefit to patients.^13^, ^14^, ^15^ These observations have generated a focus on de‐implementing low‐value care practices. The field of implementation science has grown tremendously in recent years, and the methods used for implementation are now being applied to the process of de‐implementation—a systematic approach to discontinuing the use of low‐value healthcare practices.^16^, ^17^ The optimal approach to de‐implementation should include stakeholder engagement, leadership buy‐in, and organizational readiness.^17^, ^18^ Additionally, the mechanism for de‐implementation should be simple, not requiring significant effort for the team that would be perceived as a barrier.^19^ We used these principles of de‐implementation science in order to successfully change practice at the level of a multi‐campus, regionally expansive radiation oncology department.

Interestingly, the timeline for the change from routine use to a reliable drop in diode utilization for 3D conformal radiotherapy cases was approximately 1 month from the time that the policy was enacted. This observation is in part due to patients who underwent simulation prior to our policy change but were treated after. Additionally, while our automated note process facilitated the change based on passive action versus a non‐automated approach requiring active action on behalf of the attending, nonetheless there was a need for a brief period of adaptation due to this relatively significant change in workflow, particularly for radiation therapists. This observation also highlights the importance of stakeholder buy‐in and education.

Limitations of this study include evaluation of the impact of de‐implementation within a single institution. This may reduce generalizability of this specific approach outside of our system, particularly since specific QA practices may be institution‐dependent. Nonetheless, we were able to enact the de‐implementation policy successfully across all five clinical sites within our department, which include a free‐standing community practice as well as hospital‐based practices of varying clinical volume and staff support. Success across such a range of practices reinforces the feasibility of similar de‐implementation strategies elsewhere.

Another potential criticism of this approach is that impact of this intervention may be considered of relatively low importance in the scope of the practice of radiation oncology. However, the unnecessary utilization of diodes contributes to global costs of treatment, including monetary costs, patient time, and workload for the department. When comparing the relative costs of different forms of in vivo dosimetry, Kesteloot et al. considered the detailed manner in which the additive costs of diodes must be considered when utilized for QA, including both fixed and variable costs.^20^ Such fixed costs (i.e., costs that do not vary by the number of measurements taken) include the price of the diode reader and a pair of diodes, with the assumption that the reader and the diodes have 5‐ and 2‐year average life spans, respectively. The authors estimated that the annual costs for operation and maintenance of this equipment would be approximately 5% of the reader's purchase price and assumed that each treatment unit would require its own reader, as switching between units requires recalibration. Fixed workload costs include time (and thus proportion of wages) spent performing initial and periodic calibration activities by physicists. Variable costs (e.g., costs that increase with the number of patients treated or measurements taken) relevant to diode use include consideration that more complex plans with multiple fields require an increased number of diode measurements, which can shorten the lifespan of the device. Workload variable costs for physics and radiation therapists similarly increase depending on the number of fields per plan, with time (and thus proportion of wages) required for device setup, reading, interpretation, and documentation. For each conventional 3D plan at our practice, it is estimated that a therapist spends 1 min per field on the first fraction for diode measurement. For a plan with less than 5 fractions, the therapy team will remove the diode after 10% of monitor units (MU) has been delivered in order to prevent the diode shielding effect. This process will add an additional minute to the workflow. Therefore, the cumulative impact of reduction in diode is likely meaningful on a day‐to‐day basis in our practice in terms of global reduction in the cost of treatments and more efficient utilization of our treatment machines. Finally, the benefit of unnecessary diode use extends to the patient. The dose shadowing effect of the diode is well documented, and our selective diode use also limits this dose shadowing effect to selected patient cases where a benefit of diode use is greater than the dose shadowing.^6^, ^21^

Our data demonstrate a rapid change in practice based on a simple intervention backed by stakeholder and leadership engagement, and a sound rationale for the change. Based on the success of this initiative, we anticipate conducting a similar review process of other potentially low‐value practices in our department.

## AUTHOR CONTRIBUTIONS

Serena Mao: Collection and/or assembly of data and manuscript writing. Joseph Moore: Collection and/or assembly of data and manuscript writing. Sarah Han‐Oh: Collection and/or assembly of data and manuscript writing. Ellen Huang: Collection and/or assembly of data and manuscript writing. Todd R. McNutt: Collection and/or assembly of data. Annette N Souranis: Collection and/or assembly of data. Valerie Briner: Collection and/or assembly of data. Aditya Halthore: Collection and/or assembly of data. Sarah R. Alcorn: Collection and/or assembly of data. Jeffrey J. Meyer: Collection and/or assembly of data. Akila N. Viswanathan: Collection and/or assembly of data. Jean Wright: Provision of study material and final approval of manuscript.

## CONFLICT OF INTEREST STATEMENT

The authors declare no conflicts of interest.
